# End-to-end model-based trajectory prediction for ro-ro ship route using dual-attention mechanism

**DOI:** 10.3389/fncom.2024.1358437

**Published:** 2024-02-21

**Authors:** Licheng Zhao, Yi Zuo, Wenjun Zhang, Tieshan Li, C. L. Philip Chen

**Affiliations:** ^1^Navigation College, Dalian Maritime University, Dalian, China; ^2^Maritime Big Data and Artificial Intelligent Application Centre, Dalian Maritime University, Dalian, China; ^3^Key Laboratory of Safety and Security Technology for Autonomous Shipping, Dalian Maritime University, Dalian, China; ^4^School of Automation Engineering, University of Electronic Science and Technology of China, Chengdu, China; ^5^School of Computer Science and Engineering, South China University of Technology, Guangzhou, China

**Keywords:** ship fixed route, prediction, end-to-end model, attention mechanism, DAE2ENet

## Abstract

With the rapid increase of economic globalization, the significant expansion of shipping volume has resulted in shipping route congestion, causing the necessity of trajectory prediction for effective service and efficient management. While trajectory prediction can achieve a relatively high level of accuracy, the performance and generalization of prediction models remain critical bottlenecks. Therefore, this article proposes a dual-attention (DA) based end-to-end (E2E) neural network (DAE2ENet) for trajectory prediction. In the E2E structure, long short-term memory (LSTM) units are included for the task of pursuing sequential trajectory data from the encoder layer to the decoder layer. In DA mechanisms, global attention is introduced between the encoder and decoder layers to facilitate interactions between input and output trajectory sequences, and multi-head self-attention is utilized to extract sequential features from the input trajectory. In experiments, we use a ro-ro ship with a fixed navigation route as a case study. Compared with baseline models and benchmark neural networks, DAE2ENet can obtain higher performance on trajectory prediction, and better validation of environmental factors on ship navigation.

## 1 Introduction

It is crucial to obtain dynamic information and data on ship navigation, so as to provide trajectory predictions and develop security-friendly programs and countermeasures for ships' intelligent navigation systems (Lehtola et al., 2019). Currently, navigation monitoring information in the shipping process is mainly obtained via the automatic identification system (AIS), which can record the ship number, navigation position, speed, course, and other information. AIS data can provide reliable data support for research and analysis such as maritime traffic analysis, trajectory prediction, and route planning (Zhe et al., 2020; Li et al., 2023). To predict trajectories from the perspective of ship navigation, researchers often use kinematic modeling-based methods, such as the Kalman filter, nearly constant velocity, Bayesian model, and Gaussian-sum filter, which have made good achievements in ship trajectory prediction (Mazzarella et al., 2015; Enrica et al., 2018; Baichen et al., 2019; Rong et al., 2019). The characteristics of these methods make them more suitable for ships sailing in a relatively stable environment. Navigating ships is typically impacted by several geographical conditions, which require consideration of historical data for training prediction models to enhance generalization (Gao et al., 2021). For this situation, machine learning techniques can provide higher prediction accuracy and better generalization ability compared with kinematic methods. Classic machine-learning models have been extensively utilized in the realm of ship trajectory prediction, such as logistic regression (LR) (Sheng et al., 2017), support vector machines (SVM) (Liu et al., 2019), along with many kinds of neural networks (NN) of multi-layer perceptron (MLP) (Valsamis et al., 2017), back-propagation NN (BPNN) (Simsir and Ertugrul, 2009), recurrent neural network (RNN) (Cho et al., 2014; Capobianco et al., 2021), and long short-term memory (LSTM) (Ma et al., 2022; Tang et al., 2022).

However, classic NNs lack a mechanism that can effectively mine information between sequences, so they have obvious limitations when dealing with sequential prediction problems (Zhang et al., 2022). RNN and LSTM can process sequential information, which controls the transmission of information flow through a network by adding gated mechanisms (Schmidhuber and Hochreiter, 1997; Cho et al., 2014). Zhao et al. (2023) proposed an RNN-based encoder-decoder model for trajectory prediction during ship encounter situations, where the encoder-decoder model provided improvement for handling sequential information. For this case, attention mechanisms provide a more appropriate solution (Luong et al., 2015). Several researchers have introduced the attention mechanism in the trajectory prediction model (Ma et al., 2020; Liang et al., 2022; Liu et al., 2022). Another group of researchers used attention mechanisms for feature extraction in sequential prediction. In Jiang and Zuo (2023), a multi-class trajectory prediction model was trained using the attention mechanism, and significant predictive ability was achieved in predicting the trajectory sequence. In Chen et al. (2023), an attention mechanism was applied to associate trajectory change trends with ship navigation states, and adaptively update the weighted factors of features to improve prediction accuracy.

After a review of existing studies, this article proposes a dual-attention (DA) based end-to-end (E2E) neural network (DAE2ENet) model for sequential prediction of ship trajectory. There are two mainly improved parts of the DA mechanism and E2E structure. In the E2E structure, we design a parallel network of LSTM units to extract the complex relationship between the historical and current states of ship trajectories. In the DA mechanism, we incorporate two attention mechanisms, namely global attention (GA) and local attention (LA). The GA facilitates the identification of associations between the input and output sequences, which enables the dynamic adjustment of input sequence weights to suit various prediction tasks. The LA is employed for acquiring significant characteristics from the input sequence when generating the output. In comparison experiments, traditional models (e.g., LR, SVM, BPNN), and classic NNs (e.g., RNN, LSTM, Attention) are used as baseline methods. The results show that DAE2ENet improves the accuracy by around 50% compared to the classic NNs in ship trajectory prediction. In ablation experiments, the effect of LSTM, LA, and GA are investigated, where DA can successfully capture the latent information and associations in AIS data sequences to enhance the effectiveness and generalization of trajectory prediction. According to numerical results, DAE2ENet has improved accuracy by around 30% compared to other attention models.

The remaining parts of this article are presented as follows. Section 2 presents the prosed model of dual attentions, LSTM unit, and end-to-end structure. Section 3 presents experimental results, comparisons, and validations. Section 4 presents conclusions and future plans.

## 2 Methodology

### 2.1 Variable statement of trajectory prediction

This article aims to predict the navigation position of ship trajectory based on navigating variables (*X*_*nav*_) and environmental variables (*X*_*env*_). Data gathering of navigating variables is mostly based on AIS, which includes longitude, latitude, speed, course, and so on. Data gathering of environmental variables is mostly based on sensors, which include wind, propeller pitch, rudder, and so on. Equation (1) is a set of ship navigation status and the environmental situation at time *t*.


(1) $$\begin{array}{l} x_{t}=\left\{X_{nav}\left(t\right),X_{env}\left(t\right)\right\} \end{array}$$

The current and historical navigational states have an impact on the position at sea of the ship in the upcoming moments during the sailing process. The sequence of navigation and environmental variables are shown as *X*_*nav*_ = {*X*_*nav*_(*t*), *X*_*nav*_(*t* − 1), …, *X*_*nav*_(*t* − *m*)} and *X*_*env*_ = {*X*_*env*_(*t*), *X*_*env*_(*t* − 1), …, *X*_*env*_(*t* − *m*)}, where *m* denotes the time step used in the prediction trajectory. To predict the future position of ship trajectory at time *t* + 1, the mathematical expression is formulated as Equation (2),


(2) $$\begin{array}{l} {ŷ}_{t+1}=f\left(x_{t},x_{t-1},\ldots ,x_{t-m}\right) \end{array}$$

where ŷ_*t*+1_ denotes predicted position of longitude and latitude, and *f*(·) denote the predicting function.

### 2.2 Overview of prediction framework

**Figure 1:**
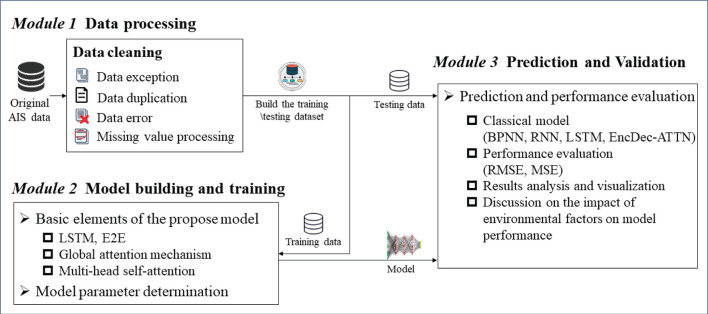
Overview of prediction framework for ship trajectory.

The proposed prediction framework of ship trajectory consists of three major segments that are shown in the diagram in Figure 1. Module 1 is data processing, which includes data cleaning for data exceptions, duplication, errors, and missing values from raw data. This process also provides training and testing data for Modules 2 and 3. Module 2 is model building and training, where DAE2ENet is trained by incorporating LSTM-based E2E structure, local attention, and global attention. Module 2 also includes the fine-tuning process of DAE2ENet parameters based on training data. Module 3 is prediction and validation, which includes comparison experiments with baseline models, and ablation experiments with proposed models.

### 2.3 Methodological design of DAE2ENet

**Figure 2:**
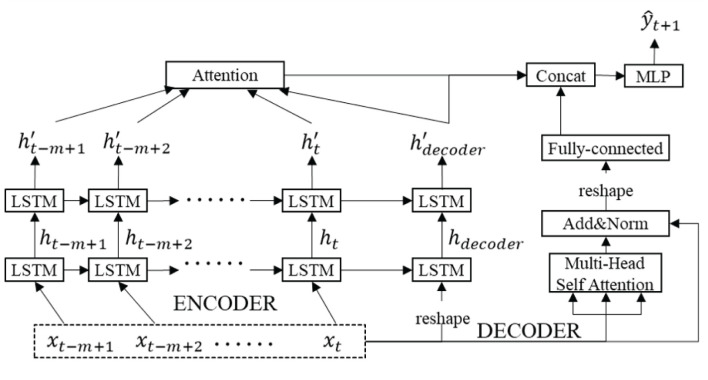
Network structure and organization of DAE2ENet.

The LSTM-based E2E structure extracts interactional information between sequences efficiently based on inputs. Since it becomes difficult to understand the dependence on information flowing control, the attention mechanism has been used to learn the dependence of input and output information. Therefore, this article proposes dual attention be incorporated into the E2E structure, where global attention is used to capture relationships from input to output, and local multi-head self-attention is used to extract dependent information in the input sequence. Figure 2 shows the visualization of the proposed model.

In the encoder block, we employ a forward network with two parallel LSTM cells in the hidden layer to pursue sequence data in the input layer. After the hidden layer, hidden states are aggregated by global attention, and dependent information with different relevant weights between input sequence and output value. In the decoder block, multi-head self-attention is employed to explore potential relationships among sequences of input information and generate representations of the relevance between input feature vectors. In the output layer, encoder states depending on global attention and local self-attention are concatenated for final output via MLP. In the encoder, the input sequence is given a new shape to the array without changing the data through the reshape operation, as input to the LSTM unit of the decoder. In the decoder, Add&Norm is used to add up the inputs and outputs for the multi-head attention mechanism and perform layer normalization operations. After Add&Norm, fully connected layer is to map the features extracted by the multi-head attention mechanism to the final output space as shown in Figure 2.

#### 2.3.1 LSTM-based E2E

**Figure 3:**
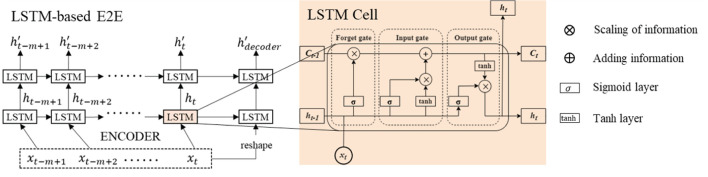
Network structure and organization of LSTM-based E2E.

The DAE2ENet reconstructs the decoder based on the LSTM-based E2E structure and combines it with dual attention mechanisms. Figure 2 shows the overall structure of the DAE2ENet model, and Figure 3 shows the structure of LSTM-based E2E as well as the operational structure of the LSTM cell. During the whole information flow of the encoding block, the LSTM transfers the input sequence into vector representation according to forward direction. LSTM is an RNN based on a gating strategy. It can effectively solve information loss caused by gradient vanishing in traditional RNNs.

- *x*_*t*_ represents the input sequence, which is given in Equation (1).
- *h*_*t*_ and $h_{t}^{\prime }$ represent the hidden state of the LSTM cell.
- ŷ_*t*+1_ represents the output sequence, which is given in Equation (2).
- The symbol σ refers to sigmoid activation function.
- *C*_*t*_ represents the cell state of the LSTM cell.

The calculation process for three gate mechanisms is listed as follows.

- Forget gate calculates the information that needs to be forgotten at time t, using the previous hidden state *h*_*t*−1_, previous cell state *C*_*t*−1_, and current input *x*_*t*_. *f*_*t*_ denotes state of forget gate as Equation (3).
  (3) $$\begin{array}{l} f_{t}=\sigma \left(\left[h_{t-1},x_{t}\right]\cdot W_{f}+b_{f}\right)\cdot C_{t-1} \end{array}$$
- Input gate calculates the information that needs to be transferred at time *t*. In the LSTM cell, there are two input gates. The first gate uses the sigmoid function in Equation (4) to map the states *h*_*t*−1_ and *x*_*t*_. The second gate also obtains the state from *h*_*t*−1_ and *x*_*t*_, which uses the tanh function as Equation (5).
  (4) $$\begin{array}{l} i_{t}=\sigma \left(\left[h_{t-1},x_{t}\right]\cdot W_{i}+b_{i}\right) \end{array}$$
  (5) $$\begin{array}{l} {\tilde{C}}_{t}=\tanh\left(\left[h_{t-1},x_{t}\right]\cdot W_{C}+b_{C}\right) \end{array}$$
  Then, cell state can be obtain according to Equation (9).
  (6) $$\begin{array}{l} C_{t}=i_{t}\cdot {\tilde{C}}_{t}+C_{t-1}\cdot f_{t} \end{array}$$
- Output gate calculates the current hidden state using the current cell state. Equation (6) shows the calculation formula for transforming state *h*_*t*−1_ and *x*_*t*_ into information *O*_*t*_. Equation (7) show current hidden state using *O*_*t*−1_ and *C*_*t*_ via tanh function.
  (7) $$\begin{array}{l} O_{t}=\sigma \left(W_{o}\cdot \left[h_{t-1},x_{t}\right]+b_{o}\right) \end{array}$$
  (8) $$\begin{array}{l} h_{t}=O_{t}\cdot \tanh\left(C_{t}\right) \end{array}$$

Finally, the brief output function calculated by the LSTM unit can be depicted as Equations (9) and (10),


(9) $$\begin{array}{l} h_{t}=LSTMCell\left(x_{t},h_{t-1},C_{t-1},\theta \right) \end{array}$$

(10) $$\begin{array}{l} h_{t}^{\prime }=LSTMCell\left(h_{t},h_{t-1}^{\prime },C_{t-1}^{\prime },\theta^{\prime }\right) \end{array}$$

where LSTMCell(·) represents a set of calculation rules for each gate mechanism. Notations θ and θ′ are the set of training parameters, which contain {*W*_*f*_, *W*_*i*_, *W*_*C*_, *W*_*o*_, *b*_*f*_, *b*_*i*_, *b*_*C*_, *b*_*o*_}.

#### 2.3.2 Design of dual-attention mechanism

**Figure 4:**
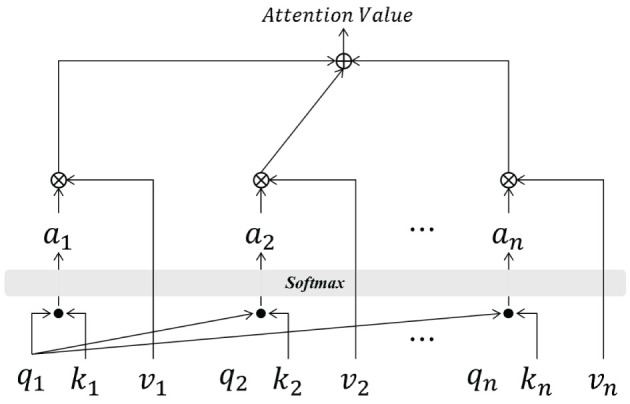
The basic calculation process of attention mechanism.

The attention mechanism has become a standard paradigm in deep learning to solve information overloading and re-allocating problems in sequential models (see Figure 4). An attention mechanism using a key-value pair is included in DAE2ENet, which contains three components: query, key, and value. The query and key vectors are calculated through dot-product to obtain the basic attention score between each current *q*_*i*_ and different *k*_*i*_, and the softmax function is used to map this score α_*i*_. The weight α_*i*_ and value *v*_*i*_ are calculated through multiplication to obtain the final attention score based on weighted summation. The calculations are given in Equations (11)–(13).


(11) $$\begin{array}{l} s\left(q_{i},k_{i}\right)=\frac{q_{i}\cdot k_{i}}{\sqrt{D}} \end{array}$$

where *D* represents the dimension of the query vector, *s*(*q*_*i*_, *k*_*i*_) represents the score function for *q*_*i*_ and *k*_*i*_.


(12) $$\begin{array}{l} \alpha_{i}=softmax\left(s\left(q_{i},k_{i}\right)\right)=\frac{exp\left(s\left(q_{i},k_{i}\right)\right)}{\overset{k}{\underset{j=1}{\sum }}exp\left(s\left(q_{j},k_{j}\right)\right)} \end{array}$$

(13) $$\begin{array}{l} att\left(\alpha_{i},v_{i}\right)=\overset{n}{\underset{i=1}{\sum }}\alpha_{i}v_{i} \end{array}$$

According to Figure 4 and Equation (13), the product of the values vector *v*_*i*_ and weight α_*i*_ obtained by Equation (12) is the attention value between the query vector and the key vector.

**Figure 5:**
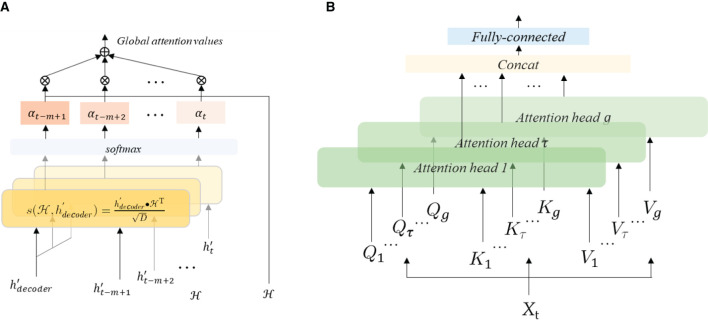
Dual-attention mechanism of DAE2ENet. **(A)** Global attention. **(B)** Local attention.

Based on the basic attention mechanism, we design dual attentions in DAE2ENet as shown in Figure 2. For the encoder, all the hidden states are inputted to calculate attention, which is considered global attention (see Figure 5A). The value of global attention is calculated by inputting the states *h*′ of the encoder and the decoding state $h_{decoder}^{\prime }$ of the decoder as H. The calculations of *A*_*global*_ are given in Equation (14)


(14) $$\begin{array}{l} A_{global}=att\left(softmax\left(H_{q},H_{k}^{T}\right),H_{v}\right) \end{array}$$

where $H_{q}$, $H_{k}$, $H_{v}$ denote the query, key, and value vectors of global attention. For the decoder, multi-head self-attention is adopted to calculate the relationships of the input sequence, which is considered as local attention (see Figure 5B). In multi-head attention calculation, a group of attention vectors *Q*_τ_, *K*_τ_, *V*_τ_ can be obtained by input *X*_*t*_, and the header value of *head*_τ_ is calculated as Equation (15)


(15) $$\begin{array}{l} head_{\tau }=att\left(softmax\left(Q_{\tau },K_{\tau }\right),V_{\tau }\right) \end{array}$$

The calculations *A*_*local*_ of all headers are concatenated as Equation (16),


(16) $$\begin{array}{l} A_{local}=concat\left(head_{1},\ldots ,head_{\tau },\ldots ,head_{g}\right)\cdot W_{MH} \end{array}$$

where *W*_*MH*_ is used for the weight parameter that can be learned during training, and *concat*(·) refers to the concatenation function, which is used to connect the outputs of multi-header self-attention.

Finally, the predicted value of ŷ_*t*+1_ can be obtain by Equation (17).


(17) $$\begin{array}{l} {ŷ}_{t+1}=MLP\left(concat\left(A_{global},FCNN\left(A_{local}\right)\right)\right) \end{array}$$

where *FCNN* denotes fully-connected neural networks, and *MLP* denotes multi-layer perceptron neural networks.

## 3 Numerical experiments

### 3.1 Data description

**Figure 6:**
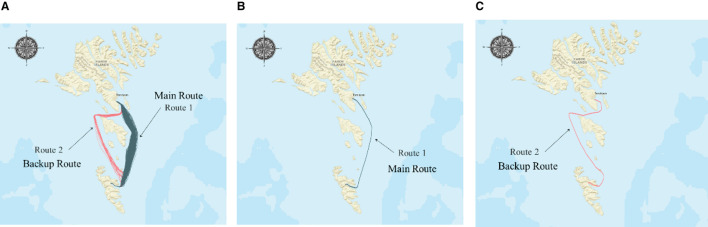
Visualization diagram of experimental trajectory. **(A)** Main route of the case ship. **(B)** Typical course of Route 1. **(C)** Typical course of Route 2.

The primary trajectory of the ship is depicted in Figure 6A. This article obtained the historical navigation trajectory from 15 February 2010 to 13 April 2010, which contains two routes. Route 1 (234 trajectories, shown in Figure 6B) is the main route, and Route 2 (38 trajectories, shown in Figure 6C) is an alternative route for worse weather conditions. The details of trajectory data are collected and displayed in Table 1.

**Table 1:** Principal information about the experimental data.

**Route**	**Longitude range**	**Latitude range**	**No. of trajectories**	**No. of points**	**Period**
Route 1	(−6.60, −6.30)	(61.30, 61.60)	234	6,135	Feb 15 to Apr 13, 2010
Route 2	(−6.60, −6.30)	(61.30, 61.60)	38	7,362	Feb 15 to Apr 13, 2010

The navigation mode of ships on the same route is consistent. Therefore, the experimental data was randomly divided for both routes 1 and 2, with 60% going toward the training set and 40% set aside for testing purposes. The experimental data comes from AIS data and onboard sensor data, and the status information is shown in Table 2. Numerical experiments discussed here involve two main components. Firstly, the main experiment is to use ship navigation factors for model training and validation. Secondly, in the discussion section, numerical experiments are conducted to explore the impact resulting from environmental factors on predicting ship trajectory.

**Table 2:** Navigation Status and environmental situation of ship trajectory.

	**Variable**	**Abbreviation**	**Sampling frequency**	**Description**
Navigation status (*X*_*nav*_)	Longitude	Lon	1 s	The ship's longitude coordinates.
	Latitude	Lat	1 s	The ship's latitude coordinates.
	Course	Cou	1 s	Course over ground.
	Speed(knots)	Spe	1 s	Speed over ground.
Environmental situation (*X*_*env*_)	Wind Angle	WA	2 s	The angle between the wind direction and the ship's heading is called the wind angle.
	Wind Speed	WS	2 s	Wind speed is the rate of airflow in the surrounding environment during ship navigation.
	Starboard propeller pitch	SPP	1 s	Propeller pitch measures forward travel per revolution.
	Port propeller pitch	PPP	1 s	
	Port Side rudder	PSR	1 s	A rudder controls a boat's direction in water, usually positioned at the stern for turning left or right.
	Starboard Side rudder	SSR	1 s	

### 3.2 Experimental preparation and setting

#### 3.2.1 Model evaluation criterion

Experimental evaluation is an important component of conducting numerical experiments. During the model training process, we chose mean squared error (MSE) as a means to quantify the disparity between the estimated and observed outcomes, and thereafter adjust the model parameters via the backpropagation method. This helps improve prediction accuracy by minimizing errors compared to the true values, thus achieving the purpose of model training. After the model training is completed, we rely on root mean square error (RMSE) metrics to measure our model output accuracy. Smaller MSE and RMSE values suggest a stronger agreement between the predicted and actual results. The Formulas of MSE and RMSE are given as Equations (18) and (19).


(18) $$\begin{array}{l} MSE=\frac{1}{p}\overset{p}{\underset{l=1}{\sum }}{\left({ŷ}_{i}-y_{i}\right)}^{2} \end{array}$$

(19) $$\begin{array}{l} RMSE=\sqrt{\frac{1}{p}\overset{p}{\underset{l=1}{\sum }}{\left({ŷ}_{i}-y_{i}\right)}^{2}} \end{array}$$

where *p* indicates sample quantity, ŷ_*i*_ denotes predicted positions and *y*_*i*_ represents real navigation positions.

#### 3.2.2 Parameter settings of the model

In the experiments, the classic optimization algorithm Adam (Kingma and Ba, 2015) is used to modify the adjustable variables in our model architecture, and the learning rate needs to be determined during operation. The sequence information encoding section of the model is frequently formed by three LSTM network structures. The hidden layer is investigated to find the optimal value within the range of [16, 320]. The magnitude of this parameter indicates the degree of non-linearity for fitting the model. When it is large, the model exhibits overfitting of the training set. For each epoch, we train 5,120 samples, which is repeated for 2,000 times. Additionally, to prevent overfitting of the model, dropout (Srivastava et al., 2014) and regularization terms were employed during the training process. Through numerous experiments, the optimized ranges, interval granularity, and optimal parameter values of the model were determined in Table 3.

**Table 3:** Basic information of model hyperparameters.

**Hyperparameters**	**Optimization boundary**	**Granularity of intervals**	**Route 1**	**Route 2**
Learning rate	(0.0001, 0.1)	0.0001	0.01	0.01
Dropout rate	(0.1, 0.5)	0.1	0.5	0.3
Number of LSTM layers	(1, 2, 3)	1	2	2
Number of hidden cells	(32, 320)	32	128	128
Regularization parameter	(0.001, 1)	0.01	0.003	0.001

#### 3.2.3 Baseline models

- LR is a continuous probability estimation method that can be used to solve regression problems when not compressing nonlinearly with a sigmoid function (Sheng et al., 2017).
- SVM determines an optimal kernel function in regression tasks, making the learned function as close as possible to predicting continuous target variables (Liu et al., 2019).
- BPNN are prevalent methods of forecasting neural networks with backpropagation (Lehtola et al., 2019).
- RNN is a classic neural network for sequential prediction (Capobianco et al., 2021).
- LSTM is one of the RNNs incorporating gating mechanisms (Tang et al., 2022).
- EncDec-ATTN is an encoder-decoder model including attention mechanism (Capobianco et al., 2021).
- DAE2ENet is the proposed method in this article.
- LAE2EDNet is one of variant DAE2ENet remaining only local attention.
- GAE2ENet is one of variant DAE2ENet remaining only global attention.
- DAE2EMLP is one of variant DAE2ENet replacing LSTM with MLP.

### 3.3 Experimental comparisons and analyses

#### 3.3.1 Comparison of model performance

In comparison with baseline models, we only use navigation status as input *X*_*nav*_ = {*X*_*nav*_(*t*), *X*_*nav*_(*t* − 1), …, *X*_*nav*_(*t* − *m*)}, where *X*_*nav*_(*t*) = {*Lon*(*t*), *Lat*(*t*), *Spe*(*t*), *Cou*(*t*)}. The output is the predicting position of ŷ(*t* + 1) = {*Lon*(*t* + 1), *Lat*(*t* + 1)}. The last column of Table 4 shows the optimal parameter values of each model during training. When SVM is used for regression prediction experiments, we chose the Gaussian radial basis (RBF) function as kernel and selected the penalty coefficient *c* = 2.1 for the objective function and the coefficient *gamma* = 0.02. For neural network models, the optimal parameter of this column represents the number of hidden layers, number of hidden units, learning rate, and regularization value, respectively. According to the results of Table 4, deep learning methods based on LSTM have achieved better performance compared to traditional methods (such as LR, SVM, and BPNN). On the other hand, models incorporating attention mechanisms performed better than baseline methods. LAE2EDNet and GAE2ENet performed worse than DAE2ENet, which reveals that incorporating the dual-attention mechanism boosts overall performance. In addition, compared with DAE2ENet, the variant model DAE2EMLP also has poor performance, indicating that when using LSTM as the encoding and decoding structure, gated mechanisms can more effectively extract information from sequential data.

**Table 4:** Comparison of model performance indicators.

	**Model**	**Route 1**		**Route 2**		**Optimal parameter**
		**MSE**	**RMSE**	**MSE**	**RMSE**	
Baseline models	LR	9.62e-4	0.0177	8.38e-4	0.0167	-
	SVM	5.76e-3	0.0413	3.62e-3	0.0345	c = 2.1, gamma = 0.02
	BPNN	7.00e-4	0.0057	4.71e-5	0.0053	2, (128, 64), 0.01, 0.002
	RNN	6.38e-4	0.0045	2.39e-5	0.0040	2, (128, 128), 0.05, 0.05
	LSTM	6.28e-4	0.0042	1.65e-5	0.0036	2, (128, 128), 0.01, 0.002
	EncDec-ATTN	2.35e-5	0.0030	1.26e-5	0.0028	2, (128, 128), 0.02, 0.001
Our models	**DAE2ENet**	9.10e-6	**0.0016**	8.35e-6	**0.0019**	2, (128, 128), 0.01, 0.003
	LAE2ENet	1.09e-5	0.0028	9.38e-6	0.0024	2, (128, 128), 0.01, 0.003
	GAE2ENet	7.10e-5	0.0023	1.05e-5	0.0027	2, (128, 128), 0.01, 0.003
	DAE2EMLP	2.40e-5	0.0041	2.29e-5	0.0039	2, (128, 128), 0.01, 0.003

The bold values mean the best prediction results can be obtained under current conditions.

#### 3.3.2 Result of ship trajectory prediction

**Figure 7:**
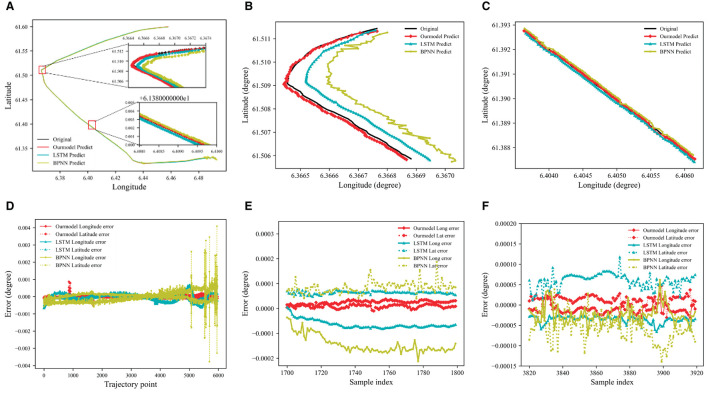
Prediction results of the trajectory for Route 1. **(A)** Model predicted results Route 1. **(B)** Prediction results of the turning phase. **(C)** Prediction results of straight stage. **(D)** Model predicted error value of Route 1. **(E)** Prediction error value of turning phase. **(F)** Prediction error value of straight stage.

The visual representation of the experimental results is depicted in Figure 7. Our model of DAE2ENet predicts a route that is consistent with the actual location of Route 1. Figure 7A displays the forecast outcomes of each model, while Figure 7D highlights discrepancies between actual and estimated movement trajectories. The overall comparison shows that predictions of longitude and latitude obtained by DAE2ENet have the smallest errors compared to other models. Figures 7B, C show more details about the performance of DAE2Net in predicting turning and straight navigation. Especially during ship turning, its prediction error gradually increases in the longitudinal direction due to changes in the navigation status of the ship. However, the prediction result of our model is more stable and has less error compared to other models. Qualitative and numeric analyses provide additional confirmation of the functionality and implementation ability.

**Figure 8:**
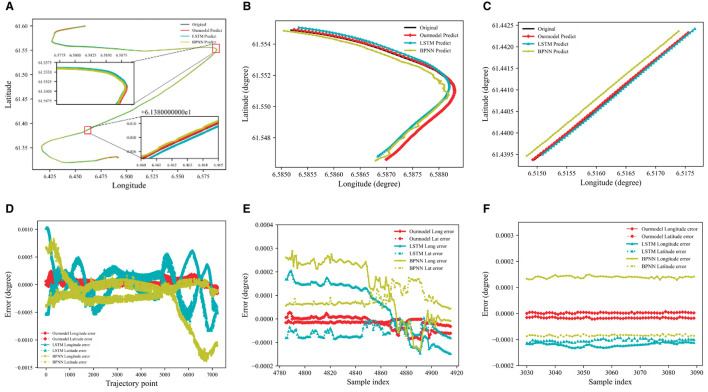
Prediction results of the trajectory for Route 2. **(A)** Model predicted results Route 2. **(B)** Prediction results of the turning phase. **(C)** Prediction results of straight stage. **(D)** Model predicted error value of Route 2. **(E)** Prediction error value of turning phase. **(F)** Prediction error value of straight stage.

Route 2 is used as validation, and the results are shown in Figure 8. Route 2 has larger turning angles, which makes prediction more difficult. As shown in Figure 8D, the prediction errors of BPNN and LSTM have greater volatility and worse prediction performance, especially during navigation turning. DAE2ENet shows lower prediction errors with smaller variations and indicates superior generality and reliability for trajectory prediction.

### 3.4 Discussion and implications

During navigation, ships are not only influenced by their navigation factors (*X*_*nav*_={*Lon, Lat, Cou, Spe*}), but also affected by environmental factors (*X*_*env*_={*WS, WA, SPP, PPP, PSR, SSR*}). In this section, we incorporated environmental factors into DAE2ENet as shown in Table 2. and investigated the effect of environmental factors on our model. The investigation data used four categories and six types of environmental factors.

In this investigation, ship navigation status *X*_*nav*_ was added sequentially to the three groups of environmental situations to explore the repercussions of external factors on maritime route predictions. The results are shown in Table 5. When Route 1 is combined with ship navigation *X*_*nav*_ and *X*_*env*_={*WS, WA*}, the RMSE value is minimal. For Route 2, the RMSE value is minimal without consideration of environmental factors. When combined with propeller pitch *X*_*env*_={*SPP, PPP*} and rudder angle *X*_*env*_={*PSR, SSR*}, the prediction accuracy of the model decreased. The findings reveal that in the actual trajectory prediction process, environmental factors are unnecessary to maintain a positive effect on the prediction efficiency.

**Table 5:** RMSE of cumulative combinations for different variables.

**Category**	**Input feature variables**	**RMSE**	
		**Route 1**	**Route 2**
*C*_1_ = {*X*_*nav*_}	{*Lon, Lat, Cou, Spe*}	0.0016	**0.0019**
*C*_2_ = {*X*_*nav*_, *X*_*env*_}	*C*_1_ ∪ {*WA, WS*}	**0.0013**	0.0030
*C*_3_ = {*X*_*nav*_, *X*_*env*_}	*C*_2_ ∪ {*SPP, PPP*}	0.0027	0.0042
*C*_4_ = {*X*_*nav*_, *X*_*env*_}	*C*_3_ ∪ {*PSR, SSR*}	0.0034	0.0051

The bold values mean the best prediction results can be obtained under current conditions.

**Figure 9:**
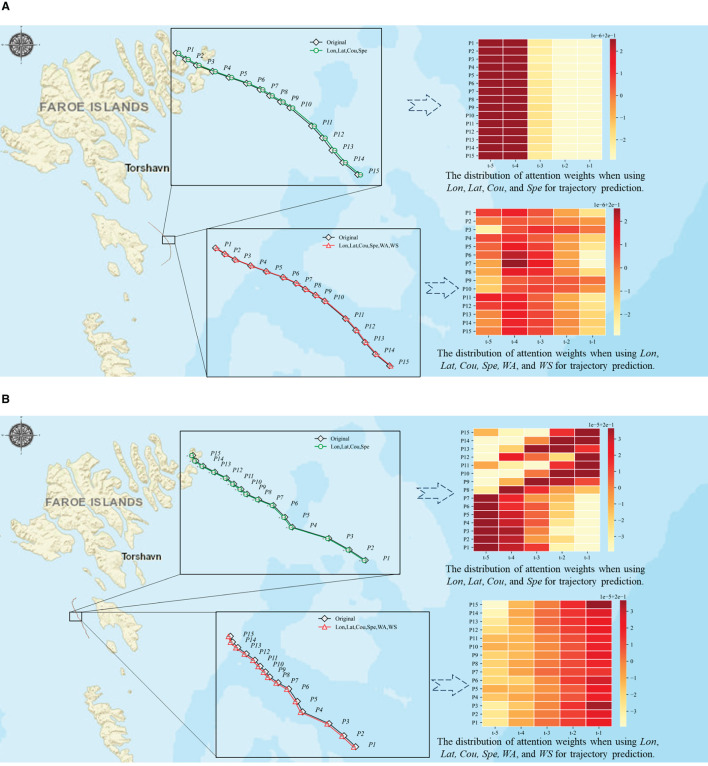
Investigation of attention weight scores in trajectory prediction for different factors. **(A)** Case of Route 1. **(B)** Case of Route 2.

To further investigate the various impacts of environmental variables regarding the prediction results of two different routes, we discussed the changes in global attention weights of the two different routes when combined with *X*_*env*_={*WS, WA*} as shown in Figure 9. In the case of Route 1 (see Figure 9A), the visual results show that the weights are changing at different positions, which helps us to improve the model's ability to predict accurately with consideration of WS and WA factors. In case of Route 2 (see Figure 9B), the distribution of attention weights at different time steps is more focused, which results in better prediction results for the model without considering WA and WS factors.

According to the comparative analysis of two sets of experiments, the attention mechanism can affect the sequence information obtained from the output by adjusting the attention weight, thereby enhancing the sequence of information related to the future and obtaining better prediction results. However, if the feature information of the input data is insufficient or unclear, the attention mechanism might lead the model to concentrate on inaccurate or irrelevant information, leading to a decrease in model performance.

## 4 Conclusions

In this article, we propose a dual-attention-based end-to-end neural network to pursue the sequential prediction task of ship trajectory. The proposed DAE2ENet introduces global attention in the encoder layer and local multi-head self-attention in the decoder layer. The global attention mechanism is employed to mine the potential relevance between input and output sequences, and the multi-head self-attention mechanism is used to capture spatial-temporal correlations among sequential feature data. Compared with previous studies, this article mainly contributes to the fields of machine learning and ship navigation from two perspectives. For technique perspective, DAE2ENet provides a novel network structure for time-series analysis and sequential prediction. From an application perspective, DAE2ENet provides a fusion process of AIS data and sensor data, and sufficiently improve the performance of trajectory prediction. Through experimental comparisons and investigations, DAE2ENet and its ablation variants outperformed baseline models including classic and state-of-art neural networks. The numerical results show that DAE2ENet improved the accuracy by around 45–70% in RMSE compared to EncDec-ATTN, LSTM, and RNN, and also obtained higher accuracy by around 30–60% in RMSE compared to LAE2ENet, GAE2Enet, and DAE2EMLP.

There are two limitations of this study. One is the impact of environmental factors on trajectory prediction. However, DAE2ENet can obtain sufficient accuracy in trajectory prediction without consideration of environmental factors. It is still necessary to improve DAE2ENet by incorporating these factors to enhance prediction accuracy. The other limitation is that dynamic navigation routes were not included to extend the applicability of DAE2ENet.

## Data availability statement

The original contributions presented in the study are included in the article/supplementary material, further inquiries can be directed to the corresponding authors.

## Author contributions

LZ: Conceptualization, Data curation, Formal analysis, Methodology, Software, Visualization, Writing – original draft. YZ: Conceptualization, Formal analysis, Methodology, Project administration, Software, Supervision, Visualization, Writing – original draft, Writing – review & editing. WZ: Funding acquisition, Project administration, Resources, Supervision, Writing – review & editing. TL: Formal analysis, Funding acquisition, Project administration, Resources, Validation, Writing – review & editing. CC: Formal analysis, Funding acquisition, Project administration, Resources, Validation, Writing – review & editing.

## References

[B1] Baichen J. Jian G. Wei Z. Xiaolong C. (2019). Vessel trajectory prediction algorithm based on polynomial fitting kalman filtering. J. Signal Process. 35, 741–746. 10.16798/j.issn.1003-0530.2019.05.002

[B2] Capobianco S. M M. L. Nicola F. Paolo B. Peter W. (2021). Deep learning methods for vessel trajectory prediction based on recurrent neural networks. IEEE Transact. Aerospace Electron. Syst. 57, 4329–4346. 10.1109/TAES.2021.3096873

[B3] Chen J. Jixin Z. Hao C. Zhao Y. Wang H. (2023). A tdv attention-based bigru network for ais-based vessel trajectory prediction. iScience 26:106383. 10.1016/j.isci.2023.106383 37063464 PMC10090245

[B4] Cho K. van Merrienboer B. Çaglar Gülçehre Bougares F. Schwenk H. Bengio Y. (2014). Learning phrase representations using rnn encoder-decoder for statistical machine translation, in 2014 Conference on Empirical Methods in Natural Language Processing (EMNLP), eds Moschitti A. Pang B. Daelemans W. (Doha: Association for Computational Linguistics), 1724–1734.

[B5] Enrica A. Paolo B. M M. L. Peter W. (2018). Detecting anomalous deviations from standard maritime routes using the ornstein–uhlenbeck process. IEEE Transact. Signal Process. 66, 6474–6487. 10.1109/TSP.2018.2875887

[B6] Gao D. Zhu Y. Zhang J. He Y. Yan K. Yan B. (2021). A novel mp-lstm method for ship trajectory prediction based on ais data. Ocean Eng. 228:108956. 10.1016/j.oceaneng.2021.108956

[B7] Jiang J. Zuo Y. (2023). Prediction of ship trajectory in nearby port waters based on attention mechanism model. Sustainability 15:7435. 10.3390/su15097435

[B8] Kingma D. P. Ba J. (2015). Adam: a method for stochastic optimization, in International Conference on Learning Representations (LCLR) (San Diego, CA), 1–15.

[B9] Lehtola V. Montewka J. Goerlandt F. Guinness R. Lensu M. (2019). Finding safe and efficient shipping routes in ice-covered waters: a framework and a model. Cold Reg. Sci. Technol. 165:102795. 10.1016/j.coldregions.2019.102795

[B10] Li Y. Liang M. Li H. Yang Z. Du L. Chen Z. (2023). Deep learning-powered vessel traffic flow prediction with spatial-temporal attributes and similarity grouping. Eng. Appl. Artif. Intell. 126:107012. 10.1016/j.engappai.2023.107012

[B11] Liang M. Liu R. W. Zhan Y. Li H. Zhu F. Wang F.-Y. (2022). Fine-grained vessel traffic flow prediction with a spatio-temporal multigraph convolutional network. IEEE Transact. Intell. Transport. Syst. 23, 23694–23707. 10.1109/TITS.2022.3199160

[B12] Liu J. Shi G. Zhu K. (2019). Vessel trajectory prediction model based on ais sensor data and adaptive chaos differential evolution support vector regression (acde-svr). Appl. Sci. 9:2983. 10.3390/app9152983

[B13] Liu R. W. Liang M. Nie J. Yuan Y. Xiong Z. Yu H.. (2022). Stmgcn: Mobile edge computing empowered vessel trajectory prediction using spatio-temporal multigraph convolutional network. IEEE Transact. Ind. Informat. 18, 7977–7987. 10.1109/TII.2022.3165886

[B14] Luong T. Pham H. Manning C. D. (2015). Effective approaches to attention-based neural machine translation, in Proceedings of the 2015 Conference on Empirical Methods in Natural Language Processing, eds Màrquez L. Callison-Burch C. Su J. (Lisbon: Association for Computational Linguistics), 1412–1421.

[B15] Ma H. Zuo Y. Li T. (2022). Vessel navigation behavior analysis and multiple-trajectory prediction model based on ais data. J. Adv. Transport. 2022, 1–10. 10.1155/2022/6622862

[B16] Ma J. Jia C. Xin Y. Xiaochun C. Wenkai L. Chunwei Z. (2020). A data-driven approach for collision risk early warning in vessel encounter situations using attention-bilstm. IEEE Access 8, 188771–188783. 10.1109/ACCESS.2020.3031722

[B17] Mazzarella F. Arguedas V. F. Vespe M. (2015). Knowledge-based vessel position prediction using historical ais data, in 2015 Sensor Data Fusion: Trends, Solutions, Applications (SDF) (Bonn: IEEE), 1–6.

[B18] Rong H. Teixeira A. Soares C. G. (2019). Ship trajectory uncertainty prediction based on a gaussian process model. Ocean Eng. 182, 499–511. 10.1016/j.oceaneng.2019.04.024

[B19] Schmidhuber J. Hochreiter S. (1997). Long short-term memory. Neural Comput. 9, 1735–1780. 10.1162/neco.1997.9.8.1735 9377276

[B20] Sheng K. Liu Z. Zhou D. He A. Feng C. (2017). Research on ship classification based on trajectory features. J. Navigat. 71, 100–116. 10.1017/S0373463317000546

[B21] Simsir U. Ertugrul S. (2009). Prediction of manually controlled vessels position and course navigating in narrow waterways using artificial neural networks. Appl. Soft Comp. 9, 1217–1224. 10.1016/j.asoc.2009.03.002

[B22] Srivastava N. Hinton G. Krizhevsky A. Sutskever I. Salakhutdinov R. (2014). Dropout: a simple way to prevent neural networks from overfitting. J. Mach. Learn. Res. 15, 1929–1958. 10.5555/2627435.2670313

[B23] Tang H. Yin Y. Shen H. (2022). A model for vessel trajectory prediction based on long short-term memory neural network. J. Mar. Eng. Technol. 21, 136–145. 10.1080/20464177.2019.1665258

[B24] Valsamis A. Tserpes K. Zissis D. Anagnostopoulos D. Varvarigou T. (2017). Employing traditional machine learning algorithms for big data streams analysis: the case of object trajectory prediction. J. Syst. Softw. 127, 249–257. 10.1016/j.jss.2016.06.016

[B25] Zhang X. Fu X. Xiao Z. Xu H. Qin Z. (2022). Vessel trajectory prediction in maritime transportation: current approaches and beyond. IEEE Transact. Intell. Transport. Syst. 23, 19980–19998. 10.1109/TITS.2022.3192574

[B26] Zhao L. Zuo Y. Li T. Philip Chent C. L. (2023). Application of an encoder–decoder model with attention mechanism for trajectory prediction based on ais data: case studies from the yangtze river of china and the eastern coast of the U.S. J. Mar. Sci. Eng. 11:1530. 10.3390/jmse11081530

[B27] Zhe X. Xiuju F. Liye Z. Mong G. R. S. (2020). Traffic pattern mining and forecasting technologies in maritime traffic service networks: a comprehensive survey. IEEE Transact. Intell. Transport. Syst. 21, 1796–1825. 10.1109/TITS.2019.2908191

